# Zooming into creativity: individual differences in attentional global-local biases are linked to creative thinking

**DOI:** 10.3389/fpsyg.2015.01647

**Published:** 2015-10-30

**Authors:** Sharon Zmigrod, Leor Zmigrod, Bernhard Hommel

**Affiliations:** ^1^Cognitive Psychology Unit, Institute for Psychological Research, Leiden UniversityLeiden, Netherlands; ^2^Leiden Institute for Brain and Cognition, Leiden UniversityLeiden, Netherlands; ^3^Department of Psychology, University of CambridgeCambridge, UK

**Keywords:** creativity, attention, individual differences, thinking and reasoning, intelligence

## Abstract

While recent studies have investigated how processes underlying human creativity are affected by particular visual-attentional states, we tested the impact of more stable attention-related preferences. These were assessed by means of Navon’s global-local task, in which participants respond to the global or local features of large letters constructed from smaller letters. Three standard measures were derived from this task: the sizes of the *global precedence effect*, the *global interference effect* (i.e., the impact of incongruent letters at the global level on local processing), and the *local interference effect* (i.e., the impact of incongruent letters at the local level on global processing). These measures were correlated with performance in a convergent-thinking creativity task (the Remote Associates Task), a divergent-thinking creativity task (the Alternate Uses Task), and a measure of fluid intelligence (Raven’s matrices). Flexibility in divergent thinking was predicted by the local interference effect while convergent thinking was predicted by intelligence only. We conclude that a stronger attentional bias to visual information about the “bigger picture” promotes cognitive flexibility in searching for multiple solutions.

## Introduction

Like an adjustable camera lens or a microscope, attention constantly zooms in and out between large objects or events and the smaller elements that comprise them. This is a reflection of the hierarchical structure of events in the world, whereby global objects are recursively constructed from local features. Although people are typically faster at detecting information at the global level than the local level (holistic vs. analytical view; Navon, 1977; Kimchi, 1992), there are also striking individual differences and situational factors that shape the perception of hierarchical stimuli. Studies have illustrated that the manner in which people allocate attention to these local or global levels is influenced by temporary states such as mood (Gasper and Clore, 2002; Huntsinger et al., 2010) or alertness (Van Vleet et al., 2011; Weinbach and Henik, 2014), as well as by factors such as age (Thomas et al., 2007), culture (Colzato et al., 2010a; Lao et al., 2013), religion (Colzato et al., 2008), and sexual orientation (Colzato et al., 2010b). Furthermore, clinical investigations have demonstrated that abnormal global processing is exhibited in clinical populations such as in schizophrenia (Carter et al., 1996; Granholm et al., 2002), severe depression (de Fockert and Cooper, 2014), obsessive compulsive disorder (Yovel et al., 2005) and cocaine users (Colzato et al., 2009). These individual differences in biases toward global or local processing appear to be stable over time (Dale and Arnell, 2013), and related to the individual’s sensitivity to the perceptual organization of gestalt laws (Poirel et al., 2008) as well as the way in which they systemize rules (Billington et al., 2008).

Of particular interest for the present study, global vs. local processing styles have been assumed to affect mental flexibility and creativity. For instance, Rowe et al. (2007) reported evidence suggesting that inducing positive mood does not only lead to the consideration of more spatially distributed visual information but also to better performance in a convergent-thinking task [the Remote Associates Task (RAT); Mednick, 1962]. These observations are consistent with the theoretical considerations of Derryberry and Tucker (1994), who postulate a direct connection between visual and conceptual attention, in the sense that the foci and integrational breadth of the two are related. Unfortunately, however, the observation that positive mood broadens the attentional scope could not be replicated in several studies (Huntsinger, 2012; Bruyneel et al., 2013). Another line of research seemed to have provided evidence suggesting that inducing global or local processing styles by means of perceptual tasks (e.g., having participants process the global or local aspect of visual stimuli) leads to a widening of the conceptual scope and the generation of more, and more creative ideas (Förster, 2012). Unfortunately, however, the article reporting some of the most relevant studies on this issue had to be retracted (Förster and Denzler, 2012), which again raises the question of how reliable the reported data are. Moreover, some of the supportive findings are relatively indirect. For instance, even if affective states can be taken to impact both attention to external stimuli and internal memory, they may do so in very different ways.

### Aim of Study

Recent studies on the possible connection between visual and conceptual attention were focusing on attentional states, with the idea that inducing a particular visual-attentional state might affect conceptual processing. In contrast, the present study was focusing on individual differences—i.e., traits rather than states. As discussed already, there is ample evidence that people differ with respect to the way they attend to and process the global and local aspects of visual information. This suggests that attentional control is affected by systematic and relatively long-lasting biases toward the global or the local aspect of visual information (Hommel and Colzato, 2010). If so, a connection between the control of visual attention and the control of conceptual attention (Derryberry and Tucker, 1994) should allow one to predict the latter from the former. In other words, the individual characteristics of processing the global and local aspects of visual stimuli should statistically predict the individual characteristics of conceptual processes.

**FIGURE 1:**
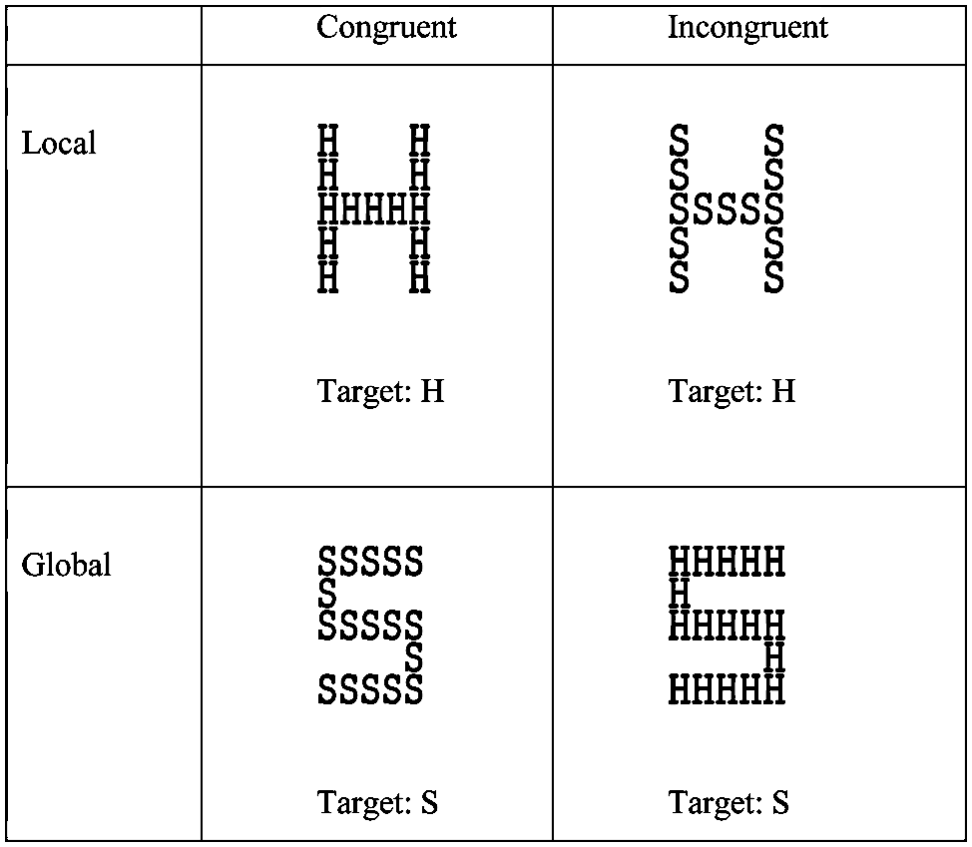
**Stimuli in the global–local task.** The participants were instructed to attend in the global block to the global level and in the local block to the local level and identify the target (“H” or “S”). The stimuli could be congruent (same letter in both levels) or incongruent (different letters for each level).

To assess the characteristics of visual attention, we employed the widely used global-local task (Navon, 1977). In this task, compound stimuli of large letters (global level) constructed from smaller letters (local level) are presented to participants, and come in two flavors; congruent, where the large and small letters are identical, and incongruent, where these differ (see **Figure 1**). Participants are instructed to focus their attention either to the large letter (global task) or the small letters (local task), and identify the correct letter (e.g., Granholm et al., 1999). This task allows for the extraction of three measures (see Navon, 1977) that we considered particularly informative regarding the individual processing style. First, the Navon task is known to produce the *global precedence effect*, i.e., people are more efficient in reacting to the global than to the local aspect of the stimuli (Navon, 1977). More interestingly for our purposes, people differ with respect to the size of this effect (e.g., Dale and Arnell, 2013), which reflects the degree to which their attentional control is biased toward the global aspect of visual stimuli. Second, performance on local aspects of stimuli is often hampered by incongruent information at the global level (e.g., if a local set of S’s is forming a global H; see **Figure 1**). We will refer to this observation as the *global interference effect* and take its size to represent the degree to which the nominally irrelevant global task set (i.e., the goal to process global information) affects local processing. Third, performance on global aspects of stimuli is sometimes hampered by incongruent information at the local level (e.g., if a global S is formed by local H’s). Given the dominance of global processing, this *local interference effect* is commonly considerably smaller than its global counterpart, suggesting that interference from incongruent stimuli is asymmetric and level-dependent (Navon, 1977). We take the size of this effect to represent the degree to which the irrelevant local task set affects global processing.

To assess the characteristics of conceptual attention we used creativity tasks (Ashby et al., 1999). While some creativity tests try to integrate various aspects of creativity, experimental studies have shown that at least some of these components are rather different and independent both theoretically and empirically (Dietrich, 2004; Hommel, 2012). In the present study, we consider the two main components, convergent and divergent thinking (Guilford, 1967). Convergent thinking consists in searching for a single solution to a well-defined problem in an analytic fashion, while divergent thinking consists in searching for many possible solutions to a vaguely defined problem (Guilford, 1967). In this study, we assessed convergent thinking by means of the RAT developed by Mednick (1962). Each item of this task is comprised of three words (such as: *boot, summer, ground*), all of which can be related to a fourth through the formation of compound words or the identification of a semantic associate (*camp*). Divergent thinking was assessed by means of the Alternate Uses Task (AUT: Guilford, 1967), in which participants are to generate as many possible uses for an everyday object such as *brick* or *newspaper*. In previous studies, performance in these two tasks was uncorrelated and differentially correlated to other aspects of cognitive performance (Akbari Chermahini and Hommel, 2010), supporting the idea that they assess orthogonal components of creativity.

Given that authors claiming a connection between visual and conceptual attention (Derryberry and Tucker, 1994; Förster, 2012) did not explicitly differentiate between convergent and divergent thinking, it is difficult to derive clear-cut predictions, but a number of expectations present themselves. Generally speaking, one would expect that an analytical thinking style goes with an attentional bias toward the local level of visual stimuli, while a more divergent thinking style should go with a bias toward the global level. If so, one would expect that RAT performance would be better for individuals with a rather small global precedence effect, which should come with little global interference but strong local interference. One would also expect that AUT performance would be better for individuals with a pronounced global precedence effect, strong global but weak local interference^1^. To test this, we had participants perform a Navon-style global-local task, a RAT, and an AUT, together with a Raven test to assess fluid intelligence—which has been shown to correlate with RAT performance (Akbari Chermahini and Hommel, 2010). The global-local task served to derive individual scores for the global-precedence effect, as well as global and local interference, which were then used to statistically predict performance in the RAT and the AUT, and vice versa.

## Materials and Methods

### Participants

In total, 124 native Dutch Leiden University students (60 men; mean age = 20 years; *SD* = 2.3 age range: 17–28 years) took part in the study for course credits or a financial reward. Three participants were excluded from the analysis, one due to misunderstanding of the divergent task, and two as a result of procedural error. All participants were right-handed with normal or corrected-to-normal vision. Exclusion criteria included: history of psychiatric disorders, drug abuse, and active medication. The study conformed to the ethical standards of the declaration of Helsinki and was approved by the Ethical Committee of Leiden University. Participants gave their written informed consent to participate.

### Stimuli and Materials

#### Global-local Task

The global-local task was modeled after Navon (1977; see **Figure 1**). In this task, participants are instructed to identify targets (“H” or “S”) either at the global level (the large letter) or the local level (the small letters that comprise the large letter) during separate experimental blocks (global block and local block). The letters can be either congruent (identical letters in the local and global levels) or incongruent (different letters in the local and global levels). The global letters were created from 5 × 5 matrices of the local letters. The height of the global letter was seven times as tall as the local letters, and both global and local letters had a ratio of 1:1.5 width to height. All stimuli were black on a light screen. Each trial began with a 500-ms tone signaling the beginning of the task followed by the stimulus that appeared in the center of the screen for 3000 ms. Participants responded by pressing on the keyboard buttons “H” or “S” with the index finger as quickly and accurately as possible. The experimental blocks were counterbalanced between subjects and prior to each experimental block; the participants read the instructions and completed four training trials. Each experimental block consisted of 72 trials.

#### Remote Association Task (RAT; Convergent Thinking)

A computerized Dutch 30-item version of the RAT was adapted from Akbari Chermahini et al. (2012; Cronbach’s alpha = 0.85). In this task, each item includes three unrelated words, and participants are asked to write a common associate as an answer (e.g., *hair, stretch, time → long*) within 30 s. After giving the solution, participants were requested to identify which problem-solving strategies they used (analytical vs. insight; cf., Bowden et al., 2005).

#### Alternate Uses Task (AUT; Divergent Thinking)

A computerized Dutch version of Guilford’s (1967) Alternative Uses Task was used. This task requires participants to list within as many possible uses for three common household items (brick, shoe, and newspaper) as possible within a span of 2 min each. Performance is scored along four measures: fluency (the total number of responses), flexibility (the number of different categories used), elaboration (the amount of detail in the responses), and originality (the amount of unusual responses). The flexibility score can be considered the theoretically most transparent and empirically most reliable of these measures (Akbari Chermahini and Hommel, 2010).

#### Raven’s Advanced Progressive Matrices Task

The Raven’s Advanced Progressive Matrices task (APM: Raven, 1965) was used to assess and estimate fluid intelligence and Spearman’s g. The task was composed of non-verbal visual patterns with one element missing. Participants choose one out of six possible answers. In this task, we used 30 items which progressively increased in difficulty over the 20 min during which the APM was administered.

### Procedure

The experiment was controlled by a Targa Pentium 3, attached to a Targa TM 1769-A 17 inch CRT monitor. Participants were tested in a small cubical room, and they were instructed to sit upright on a wooden chair and look at a fixation point. The experimenter ensured that participants faced the monitor at a distance of about 60 cm with the same visual angle. The participants read and signed the informed consent form before the beginning of the experiment. All the participants completed the four tasks. Half of the participants completed the creativity tasks (RAT and AUT) first and half of the participants completed the global-local task first. The creativity tasks were also counterbalanced between participants. The Raven task was performed last.

## Results And Discussion

### Statistical Analysis

To investigate the relationship between global–local attentional biases and creative thinking styles, performance on each task was calculated per participant. For the global-local task, mean response time of correct responses and accuracy were calculated separately for each block (global vs. local) and condition (congruent vs. incongruent). An ANOVA was performed to confirm that basic findings could be replicated (see below). For the correlation and regression analyses, various scores were calculated. Global precedence effects were computed by subtracting the mean reaction time (RT) for trials in the global task from the mean RT for trials in the local task. Global and local interference effects were computed by subtracting the RTs in congruent trials from those in the incongruent trials, separately for the local and the global task. As a measure of general response speed, we computed the average over both tasks. For the RAT and the Raven task, we calculated the number of correct items. For the AUT, two independent judges scored fluency, flexibility, and elaboration. Originality was calculated through a set of functions where each response is compared to the total amount of responses for that item from all participants. Pearson correlation coefficients were computed for all combinations of scores. **Table 1** provides an overview, for a detailed presentation of the findings see below.

**Table 1:** Correlations between global–local measurements in directed attention condition and creative style.

	Global precedence	Global interference	Local interference	Raven’s matrices	RAT	Fluency	Elaboration	Flexibility	Originality
RT overall	0.044	-0.153	0.127	-0.091	-0.221^∗^	-0.029	-0.035	-0.152	-0.015
Global precedence		0.338^∗∗^	-0.279^∗∗^	-0.013	0.089	0.056	0.093	0.112	0.137
Global interference			0 .043	0.093	0.242^∗∗^	0.066	0.075	0.131	0.114
Local interference				0.101	0.037	-0.070	-0.136	-0.198^∗^	-0.090
Raven’s matrices					0.237^∗∗^	0.010	0.071	-0.020	0.004
RAT						0.117	-0.091	0.096	0.054
AUT fluency							0.156	0.801^∗∗^	0.829^∗∗^
AUT elaboration								0.391^∗∗^	0.399^∗∗^
AUT flexibility									0.795^∗∗^


*N = 121, ^∗^p < 0.05, ^∗∗^p < 0.01.*

### Global Precedence and Global/Local Interference Effects

**FIGURE 2:**
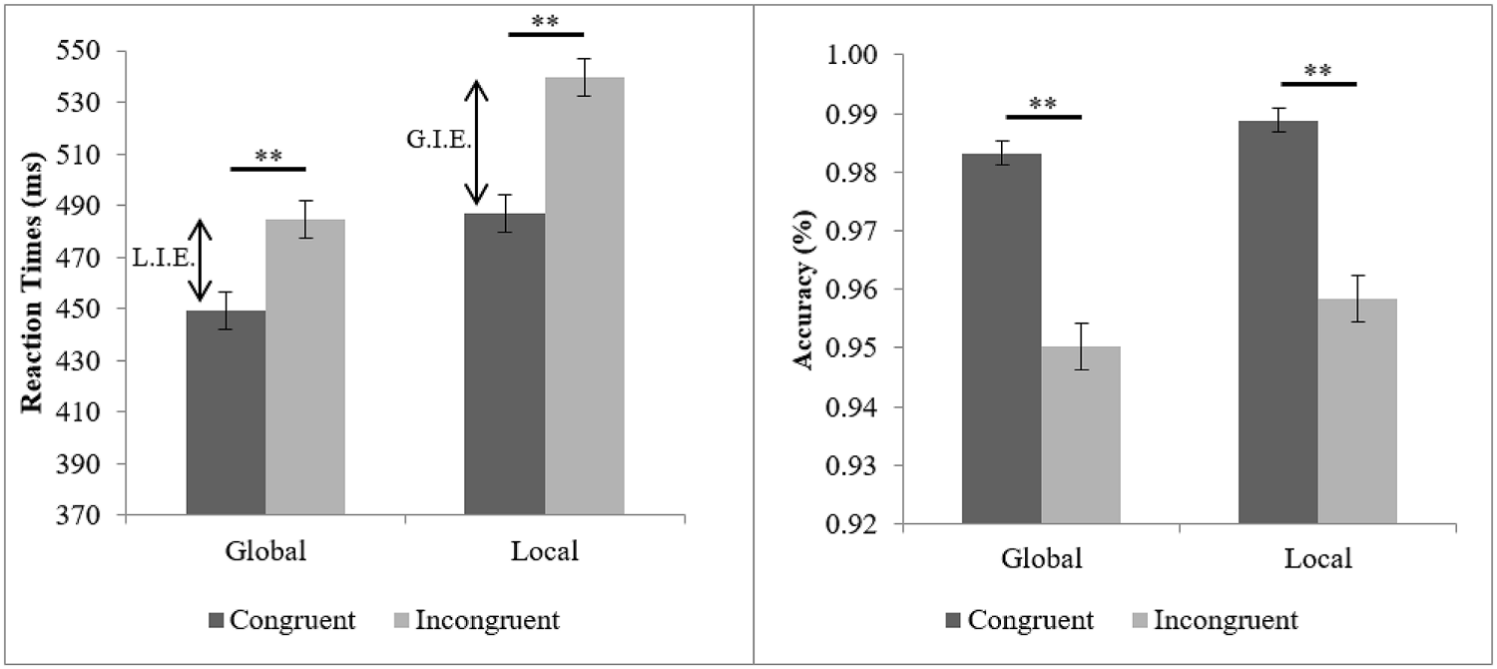
**Mean reaction times (RTs; with standard error bars) as a function of task (global vs. local) and stimulus congruency (congruent vs. incongruent).** LIE, Local interference effect; GIE, Global interference effect. ^∗∗^*p* < 0.001.

As a manipulation check, we tested whether the well-established effects of the global–local task could be replicated. Mean RTs and accuracy were analyzed by repeated measures ANOVAs as a function of the task (attending to global vs. local level) and stimulus congruency (congruent vs. incongruent) as within-subjects factors. Main effects of task, in RT, *F*(1,120) = 62.35, *p* < 0.0001, $\eta_{p}^{2}$ = 0.342, and accuracy *F*(1,120) = 5.99, *p* < 0.05, $\eta_{p}^{2}$ = 0.048, indicating faster and more accurate responses to global targets than to local targets (see **Figure 2**), replicated the global precedence effect (Navon, 1977; Kimchi, 1992). In addition, main effects of stimulus congruency in RTs *F*(1,120) = 363.83, *p* < 0.0001, $\eta_{p}^{2}$ = 0.752, and accuracy *F*(1,120) = 93.93, *p* < 0.0001, $\eta_{p}^{2}$ = 0.439, were observed. These effects replicated the global and local interference effects (see **Figure 2**). Furthermore, there was a significant interaction between task and stimulus congruency in RTs, *F*(1,120) = 16.12, *p* < 0.0001, $\eta_{p}^{2}$ = 0.118, indicating that the global interference effect (global interference in local task, GI = 52.95) was larger than the local interference effect (local interference in global task, LI = 35.19; see **Figure 2**). The correlations between the measures from the global–local task also provide a coherent picture (see **Table 1**). As one would expect, interference from the global level correlates positively with the size of the global precedence effect, which again is negatively correlated with interference from the local level.

### Predicting Convergent Thinking (RAT)

Performance on the RAT was significantly correlated with three scores: First, the positive correlation with the Raven score confirms earlier observations that fluid intelligence predicts RAT performance (Akbari Chermahini and Hommel, 2010). Second, the convergent-thinking score was positively correlated with global interference, indicating that more interference from the global level on local performance went along with better convergent thinking performance. Note that this is opposite to what we expected, as we hypothesized that RAT performance would be better for individuals with a small global precedence effect, accompanied by weak global but strong local interference. The third significant correlation gives a hint toward a possible explanation. We can see that RAT performance is negatively correlated with the general RT level. Follow-up analyses showed that the global-precedence effect was negatively correlated with the RT level in the global task, r = -0.34, *p* < 0.001, but positively correlated with the RT level in the local task, r = 0.41, *p* < 0.001. To test whether the actually expected pattern would be more apparent if only trials with analytical solutions are considered (Bowden et al., 2005), we reran the analyses after eliminating all data from trials with intuitive solutions. However, this merely rendered all correlations insignificant, *ps* > 0.23, presumably due to the data loss and the resulting increase in intra-individual variability.

Taken together, this pattern suggests the following possibility: The observation that faster participants produce stronger precedence effects implies that overcoming the dominant global bias takes time. If so, the impact of the global bias on performance in the local task decreases over time, so that faster reactions to local stimulus aspects suffer more from incongruent global information than slower reactions do. A similar temporal dynamic has been observed for the Simon effect, which is also more pronounced for fast than for slower reactions (Hommel, 1993). If this scenario applies, it follows that the correlation between convergent-thinking performance and global interference does not reflect any commonalities between visual and conceptual attention. Rather, it seems to be due to that people who are fast in the global-local task (and therefore happen to suffer more from global interference) are also good convergent thinkers. This would fit with the positive correlation of convergent thinking and fluid intelligence, which also has been shown to correlate positively with general response speed (Jensen, 1998).

### Predicting Divergent Thinking (AUT)

**FIGURE 3:**
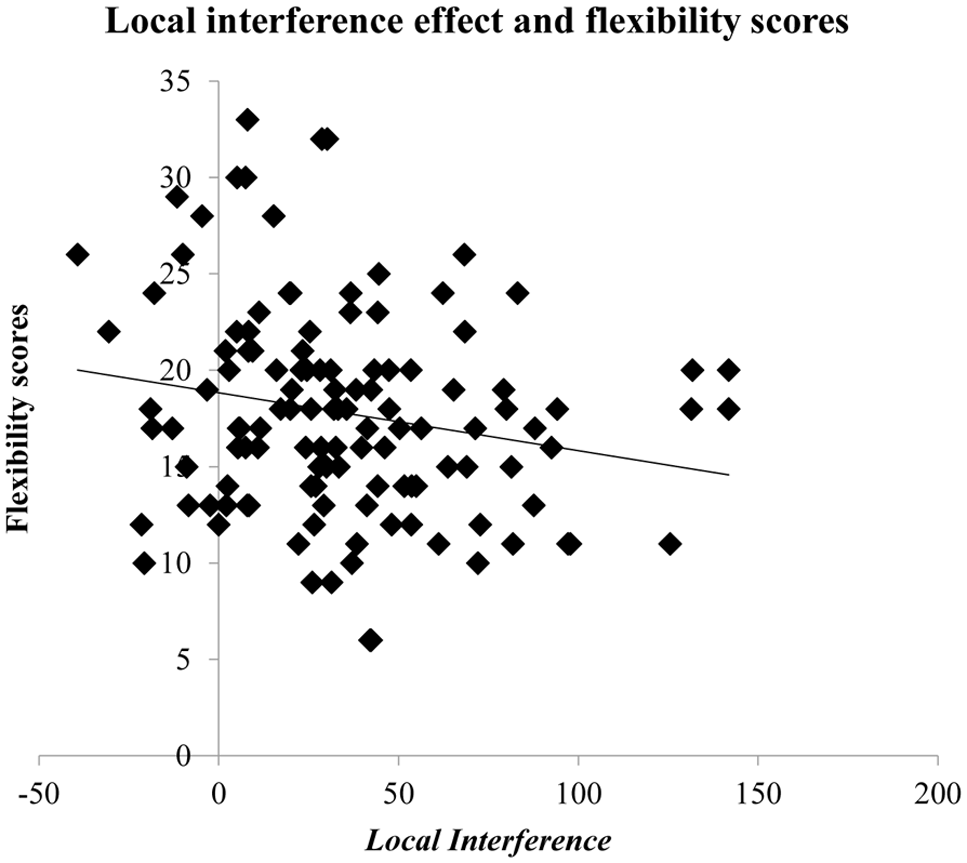
scattered plot depicting the negative correlation between the local interference effect and the flexibility scores from the divergent thinking task (AUT).

**Table 1** shows that the four scores derived from the AUT are strongly intercorrelated but that the only score that correlates with other measures is flexibility. This is consistent with previous observations, which also found this score to be the most systematic and replicable (Akbari Chermahini and Hommel, 2010). We see that flexibility is negatively correlated with local interference, indicating that better performance in the AUT comes with a weaker impact from irrelevant local information (see **Figure 3**). This observation fits with our expectations: the brainstorming-like divergent-thinking task should benefit from a more global bias rather than from attention to detail. While this did not lead to a significant positive correlation between flexibility and the global-precedence effect (which, however, goes in the right direction), it did yield the expected reduced impact from the local level.

As suggested by one of the reviewers, in order to ensure that our counterbalancing in the global–local task as well as RT did not contribute to the individual differences predicting the RAT and the Flexibility, a two-stage liner regression was performed on both RAT and Flexibility as the dependent variables and task order and RT in the first stage and global–local effects as the independent variable in the second stage. The correlational findings were not affected by task order and RT (see **Table 2**).

**Table 2:** Results of linear regression analyses for RAT scores and Flexibility scores with task order and RT as first step of the linear regression and global and local interferences as the second step of the linear regression.

	RAT scores			Flexibility scores		
		
	*B (SE B)*	β	*t*	*B (SE B)*	β	*t*
**Step 1**						
Task order	0.635 (0.60)	0.095	1.058	-0.776 (0.988)	-0.072	-785
RT	-0.009 (0.004)	-2.11	-2.342^∗^	-0.011 (0.006)	-0.160	-1.753
**Step 2**						
Task order	0.632 (0.590)	0.095	1.07	-0.766 (0.972)	-0.071	-0.788
RT	-0.008 (0.004)	-0.185	-2.05^∗^	-0.008 (0.006)	-0.117	-1.278
Global interference	0.020 (0.009)	0.210	2.358^∗^	0.019 (0.014)	0.122	1.352
Local interference	0.005 (0.008)	0.053	0.594	-0.029 (0.014)	-0.189	-2.097^∗^


*N = 121, ^∗^p < 0.05 R^2^ = 0.058 for step 1; *R*^2^ = 0.105 for step 2 (*p*s < 0.05) *R*^2^ = 0.028 for step 1; *R*^2^ = 0.075 for step 2 (*p*s < 0.05)*

### Gender Differences

We carried out additional explorative analyses to identify possible gender effects. However, RTs and accuracy in the global–local task did not differ between males and females, as revealed by one-way ANOVAs with gender as a between-subjects factor, all *p*s > 0.05, replicating previous findings (Kimchi et al., 2009). The same was true for overall RAT scores, the four AUT scores, and Raven’s Matrices scores, all *p*s > 0.05.

## Conclusion

The aim of the study was to explore possible links between core functions of attention and creativity. Using the global–local paradigm (Navon, 1977), we observed that attention allocation biases to particular levels of hierarchical stimuli can predict one’s performance characteristics in some aspects of creative thinking. Importantly, we found that convergent and divergent thinking, the two components of human creativity that we considered, were related to characteristics of performance in the global–local task in very different ways. This suggests that all creativity tasks should not be considered the same, and it also raises doubts in attempts to integrate different factors into one measure—as various creativity tests have tried.

More specifically, we found that the local interference effect was a reasonably good predictor of divergent thinking performance, at least with respect to the most transparent score flexibility. This suggests that individuals whose attention was not significantly diverted by the irrelevant local elements (the smaller letters) of the hierarchical stimulus while attending to the global aspect (the larger letter) were more likely to find varied and wide-ranging solutions to a given problem. That is, a stronger bias to the bigger picture with respect to visual events lends itself to greater cognitive flexibility in searching for multiple solutions in the divergent thinking task. It is interesting to note that studies of populations exhibiting diminished cognitive flexibility have found the reverse pattern: here, the local interference effect was *positively* correlated with obsessive-compulsive cognitive style (Yovel et al., 2005) and the effect was significantly more pronounced in individuals with autism and Asperger’s syndrome in comparison to controls (Rinehart et al., 2000; Muth et al., 2014). Furthermore, individuals displaying high systemizing tendencies have also shown greater susceptibility to local interference (Billington et al., 2008). Taking together these findings and the present results suggest that individual variability in the local interference effect may be used as an index for cognitive flexibility. High values of the local interference effect might be taken to denote rigid, narrow, obsessive-compulsive tendencies, whereas low values reflect enhanced flexibility and a capacity for divergent thinking. More research into the possibility of the local interference measure as an index for cognitive flexibility is needed, however.

In contrast to the divergent-thinking task, no systematic connection between visual and conceptual attention emerged from the convergent-thinking task. While there was a correlation between convergent-thinking performance and the global interference effect, the sign of the effect and the overall pattern including measures of general response speed strongly suggest that this correlation does not reflect mechanistic commonalities between processes underlying performance in the global–local task and the RAT. There was also no indication of a possible connection to the global-precedence effect and local interference. Taken altogether, this suggests that the RAT may not be suitable for identifying relationships between visual and conceptual attention.

Although much remains to be learned about possible connections between visual and conceptual attention, there are hints toward a shared neurobiological basis for global/local processing and divergent/convergent thinking. With respect to attentional processing, neuropsychological studies demonstrate that right hemisphere damage often leads to impairments in global processing whereas left hemisphere lesions can disrupt local processing (Delis et al., 1986; Robertson et al., 1988; Lamb et al., 1990). There is also evidence from imaging studies supporting this hemispheric asymmetry (Fink et al., 1996; Volberg and Hübner, 2004; Gable et al., 2013). Interestingly, comparable patterns are also emerging in the study of creative thinking styles. In spite of the complexities associated with neuroimaging research into creativity (Arden et al., 2010), neurostimulation experiments are beginning to reveal a similar hemispheric lateralization in creativity. It has been illustrated that convergent thinking can be enhanced by stimulating the left prefrontal cortex with anodal transcranial direct current stimulation (Cerruti and Schlaug, 2009; Metuki et al., 2012; Zmigrod et al., in press), and complementarily, divergent thinking performance can be improved by anodal tDCS over right frontal regions (Mayseless and Shamay-Tsoory, 2015). These parallels could suggest that zooming into the brain could provide a fruitful basis for future research into the links between attentional processing biases and creative thinking styles.

## Conflict of Interest Statement

The authors declare that the research was conducted in the absence of any commercial or financial relationships that could be construed as a potential conflict of interest.

## Footnotes

1 Note that it is difficult to derive precise predictions regarding the relationship between global and local interference. One problem is that global interference is commonly more pronounced than local interference (Navon, 1977), which implies that individual local-interference scores rely on lesser variability than global-interference scores–which is a problem for correlational analyses. Indeed, we will see that the two measures did not correlate significantly in our study. Another, but related problem is that the relative size of the two scores for a given individual is likely to be mediated by his or her degree of global precedence. Individuals with considerable global precedence are likely to have large global-interference scores but small local-interference scores, and the opposite is true for individuals with a small global-precedence effect. This means that estimates of global and local interference are likely to differ in reliability for each given individual. As a consequence, we did not try to present separate predictions for global and local interference and their associations with other measures, but considered the possibility that (depending on the role of global precedence) some associations may express themselves through correlations with global interference scores while other associations may have a stronger impact on local interference scores.
